# Isolation and Identification of Lactic Acid Bacteria from Natural Whey Cultures of Buffalo and Cow Milk

**DOI:** 10.3390/foods11020233

**Published:** 2022-01-16

**Authors:** Rosangela Marasco, Mariagiovanna Gazzillo, Nicoletta Campolattano, Margherita Sacco, Lidia Muscariello

**Affiliations:** Dipartimento di Scienze e Tecnologie Ambientali, Biologiche e Farmaceutiche, Università degli Studi della Campania “L. Vanvitelli”, Via Vivaldi 43, 81100 Caserta, Italy; rosangela.marasco@unicampania.it (R.M.); maryj7291@gmail.com (M.G.); nicoletta.campolattano@unicampania.it (N.C.); margherita.sacco@unicampania.it (M.S.)

**Keywords:** natural whey starter, lactic acid bacteria, caciocavallo cheese, mozzarella cheese, microbial diversity

## Abstract

In southern Italy, some artisanal farms produce mozzarella and caciocavallo cheeses by using natural whey starter (NWS), whose microbial diversity is responsible for the characteristic flavor and texture of the final product. We studied the microbial community of NWS cultures of cow’s milk (NWSc) for the production of caciocavallo and buffalo’s milk (NWSb) for the production of mozzarella, both from artisanal farms. Bacterial identification at species and strain level was based on an integrative strategy, combining culture-dependent (sequencing of the 16S rDNA, species/subspecies-specific Polymerase Chain Reaction (PCR) and clustering by Random Amplified Polymorphic DNA-Polymerase Chain Reaction (RAPD-PCR) and culture-independent (next-generation sequencing analysis, NGS) approaches. Results obtained with both approaches showed the occurrence of five species of lactic acid bacteria in NWSb (*Lactococcus* *lactis* subsp. *lactis*, *Lactobacillus* *fermentum*, *Streptococcus* *thermophilus, Lactobacillus* *delbrueckii*, and *Lactobacillus helveticus*) and five species in NWSc (*Lc.* *lactis* subsp. *lactis*, *Enterococcus* *faecium*, and *S.* *thermophilus*, *Lb. helveticus*, and *Lb. delbrueckii*), with the last two found only by the NGS analysis. Moreover, RAPD profiles, performed on *Lc. lactis* subsp. *lactis* different isolates from both NWSs, showed nine strains in NWSb and seven strains in NWSc, showing a microbial diversity also at strain level. Characterization of the microbiota of natural whey starters aims to collect new starter bacteria to use for tracing microbial community during the production of artisanal cheeses, in order to preserve their quality and authenticity, and to select new Lactic Acid Bacteria (LAB) strains for the production of functional foods.

## 1. Introduction

Cheese is a very valuable food product for its high nutrition value, its versatility in the end use, and its organoleptic properties. Cheese counts a wide variety worldwide, and undergoes numerous classifications based on different parameters, either depending on intrinsic characteristic of the raw material, or depending on the technic used for the production [1]. Mostly, a large variety of different cheeses are produced by the use of standardized procedures involving the addition of starter lactic acid bacteria (SLAB); during ripening, growth of non-starter lactic acid bacteria (NSLAB) occurs, depending on the microbiota of the raw milk used and the standard procedures adopted [2,3,4].

Mozzarella and caciocavallo cheeses belong to the class of Pasta filata cheese, which is based on stretching of the curd in hot water to obtain the smooth texture of the final product [5]. Originally produced in southern Italy, their market, especially for mozzarella, expanded in many countries, particularly in the USA [6]. In spite of the development of different standard procedures for industrial manufacturing of Pasta filata cheese, the production of mozzarella and caciocavallo in Campania (southern Italy) is still largely based on artisanal activities of small dairy farms. Mostly, artisanal production of mozzarella and caciocavallo uses natural whey starters (NWS). The microbial consortia of the NWS together with that of the raw milk used as a starting material account for the variety of different characteristics and quality of the final product [7,8,9,10]. Generally, the microbial biodiversity of NWSs is higher than that present in SLABs. Indeed, the microbiota of various artisanal mozzarella and caciocavallo cheeses has been determined by different molecular methods based on culture-dependent or -independent approaches [11,12,13,14,15,16]. The culture-independent metagenomic analysis has also been applied to studies on Pasta filata cheeses acidified with either SLAB or with NWS [17,18,19,20]. This approach overcomes the problem of underestimation of the microbial diversity, which is often encountered when culture-dependent methods are used. Nevertheless, isolation and characterization of lactic acid bacteria (LAB) inhabiting the NWS is important to define the specific microbiota in order to maintain high standard of reproducibility of the final product. In fact, the characteristic presence of defined LAB in NWS, together with that in raw milk, is required for specific nutritional, organoleptic and physical properties of the derived cheese (3, 5, 8). Moreover, the search for LAB isolates with probiotic characteristic is still of great value due to scientific evidence, accumulated especially in the past decade, showing gut-derived effect of probiotic LAB strains on human intestinal health [21]. Furthermore, the importance of the microbiota in the gut–brain axis is now largely explored [22,23]. Therefore, on the light of the assessed value of some LAB strains, studies on isolates from NWS may also be functional to the implementation of new promising probiotic strains.

Here, we show the taxonomic identification of the microbial species isolated from natural whey starter cultures (NWS) of cow’s milk for the production of caciocavallo, and of buffalo’s milk for the production of mozzarella, both collected from two dairy artisanal factories located in Campania region (southern Italy).

In addition, the combined use of culture-dependent (sequencing of the 16S rDNA, specie/subspecies-specific PCR (Polymerase Chain Reaction) and clustering by RAPD-PCR (Random Amplified Polymorphic DNA-Polymerase Chain Reaction) and culture-independent approaches (next-generation sequencing analysis) was applied to perform a comparative analysis of the microbial diversity at species and strain level. 

## 2. Materials and Methods

### 2.1. Natural Whey Starter Cultures Sampling

Samples of natural whey starter cultures (NWS) of cow’s milk for the production of caciocavallo (NWSc), and of buffalo’s milk for the production of mozzarella (NWSb) were collected from two dairy artisanal factories located in Campania region (southern Italy). NWS cultures were supplied in duplicate and stored at 4 °C for a few hours before analysis or at −80 °C, after the addition of 20% (*v*/*v*) glycerol, for later analysis.

### 2.2. Lactic Acid Bacteria Enumeration and Isolation

Serial decimal dilutions of NWS cultures samples were prepared in NaCl 0.9% solution. The cell suspensions were plated and incubated as follows: (I) on the Man, Rogosa, and Sharpe (MRS) agar, incubated aerobically and anaerobically for 48 h at 30 and 44 °C, for mesophilic and thermophilic rod LAB, respectively; (II) on ESTY agar, incubated aerobically and anaerobically for 48 h at 30 and 44 °C, for mesophilic and thermophilic cocci LAB, respectively; (III) on Brain Heart Infusion (BHI) agar, incubated aerobically for 48 h at 37 °C, for enterococci. Five to ten colonies of each different growth condition were isolated from the highest plate dilution by double streaking on agar media. All cultures were stored at −80 °C in 20% (*v*/*v*) glycerol.

### 2.3. Genotypic Identification by Partial 16S rDNA Gene Sequence Analysis

Genomic DNA extraction from 100 isolates was carried out using GenElute™ Bacterial Genomic DNA kit (Sigma-Aldrich, Merck group, Milano, Italy ) according to the manufacturer’s instructions. The primer pairs Gray28f (TTTGATCNTGGCTCAG) and Gray519r (GTNTTACNGCGGCKGCTG) were used to amplify the V1-to-V3 region of the 16S rDNA gene (amplicon 520 bp). Fifty microliters of each PCR mixture contained 100 ng of template DNA, 0.5 µM of each primer, 0.2 mM of each dNTP (2′-deoxynucleoside 5′-triphosphate), 2.5 mM MgCl2, 5 µL of 10X PCR buffer, and 2U of *Taq* DNA polymerase (Microtech, Naples, Italy). The following PCR conditions were used: 94 °C for 2 min, 35 cycles of 95 °C for 20 s, 56 °C for 45 s, and 72 °C for 3 min, and a final extension at 72 °C for 7 min [24]. The PCR product of each isolate was eluted and purified from gel by QIAquick Gel Extraction Kit (Qiagen, Milano, Italy). Sequencing with primers Gray28f and Gray519r was performed on both strands at Microgem, Federico II University of Naples, Italy (www.microgem.it, accessed on 17 November 2021). Taxonomic identification at species level was performed by comparing the V1-V3 sequence of each isolate with those reported in the basic BLAST database [25]. The species was assigned to the isolates showing V1-V3 sequence identity of 97% or higher. Each taxonomic assignment was confirmed by species- and subspecies-specific PCR.

### 2.4. Species- and Subspecies-Specific PCR

The synthetic primers used in species- or subspecies-specific PCR are reported in Table 1. Genomic DNA was isolated as above described. PCR amplifications were carried out by using a SimpliAmp thermal cycler (Applied Biosystems, Waltham, Massachusetts, USA) in a total volume of 25 µL, containing 50 ng of template DNA, 1X PCR buffer, 1.5 mM MgCl2, 0.5 µM of each primer, 0.2 mM of each dNTP, and 1U of *Taq* DNA polymerase (Microtech, Naples, Italy). PCR conditions were different for the various species as described in Table 2. PCR products were analyzed by electrophoresis on appropriate concentrations of agarose gel (1.5% for amplicons ranging from 200 to 600 bp, and 1% for amplicons of about 1800 bp), followed by ethidium bromide staining. The specificity of primers was evaluated by PCR of one reference strain from laboratory collection and negative controls were included as well.

### 2.5. Random Amplified Polymorphic DNA-Polymerase Chain Reaction (RAPD-PCR) Analysis

Genomic DNA from each strain was extract as above described. Four different primers were individually employed: RAPD1 (5′-AGCAGGGTCG-3′), RAPD2 (5′-AGCAGCGTCG-3′), XD9 (5′-GAAGTCGTCC-3′), and M13 (5′-GAGGGTGGCGGTTCT-3′) [12,30]. RAPD reaction mixture and amplification programs were performed as described by Fontana et al. [30]. PCR products were separated by electrophoresis at 100 V for 4 h on 2.5% agarose gel, stained with ethidium bromide (0.5 mcg mL^−1^) and visualized by UV detection (Typhoon Biomolecular Imager, Amersham, UK). The molecular weight of the amplified DNA fragments was estimated by comparison with 1 kb Plus-DNA Markers (abm, Richmond, BC, Canada) ranging from 100 to 10,000 bp. For detecting similarity among the isolated strains, only amplified fragments unambiguously present, both intensely and weakly stained, were scored as present (1) or absent (0) to form a binary matrix which was computed using Jaccard’s coefficient and used to produce UPGMA-based similarity matrix and dendrogram with DendroUPGMA (http://genomes.urv.es.UPGMA, accessed on 17 November 2021).

### 2.6. High-Throughput Sequencing

Microbial DNA extraction from both NWSc and NWSb was performed using GenElute™ Bacterial Genomic DNA kit (Sigma-Aldrich) according to the manufacturer’s instructions with slight modifications. One mL of NWS was mixed with 2% (wt/vol) of sodium citrate solution in 10 mL final volume and homogenized by Stomacher (Stomacher 400 Circulator). The mixtures were centrifuged at 10,000 rpm for 10 min at 4 °C. The supernatant was removed, and the cells were washed two times with 1 mL of sodium citrate. The cell suspension was centrifuged at 10,000 rpm for 10 min at 4 °C. Subsequently, the manufacturer’s protocol for DNA extraction from Gram positive bacteria was adopted. The concentration and purity of the extracted nucleic acids were determined by Nanodrop (NanoDrop™ 2000, Thermo Fisher Scientific, Waltham, MA, USA). Next-generation sequencing analysis was performed by BMR genomics srl service (www.bmr-genomics.it, accessed on 17 November 2021, Padova, Italy). The microbial diversity was studied by pyrosequencing of the amplified V3-V4 region of the 16S rDNA. The amplicon pools, obtained from each NWS, were purified with the Agencourt AMPure XP kit (Beckman Coulter, Milan, Italy) and re-amplified including universal primers tailed with Illumina barcode adapters. Amplicons were used for pyrosequencing on the Illumina MiSeq platform (Illumina Italy s.r.l., Milan, Italy) with 2 × 300 bp paired end approach. Sequences were analyzed by using Usearch v.10 software. Chimera filtering and OTU clustering were obtained by UPARSE-OTU using the database 16S RDP training set 17.

## 3. Results

### 3.1. Enumeration and Isolation of Lactic Acid Bacteria from Natural Whey Starter Cultures

Characterization of the microbiota of natural whey starter cultures of cow’s milk for the production of caciocavallo (NWSc) and of buffalo’s milk for the production of mozzarella (NWSb) has been performed growing the whey samples on media formulated for lactobacilli, lactococci/streptococci, and enterococci.

In NWSc, streptococci, lactococci, and enterococci were found prevalent (6.5 log CFU mL^−1^) compared to lactobacilli, ranging from 4.7 (thermophilic) to 5.6 (mesophilic) log CFU mL^−1^ (*p* < 0.05) without significant differences between mesophilic and thermophilic, whereas among lactobacilli, the mesophilic species were 1 log more abundant compared to thermophilic species. Contrariwise, in NWSb microbial groups are all equally represented, with a slightly higher concentration of thermophilic (8.5 CFU mL^−1^) and a lower concentration of mesophilic lactobacilli (7.0 CFU mL^−1^) (Table 3). No significant differences were found upon incubation in aerobic or anaerobic conditions for either NWSc or NWSb samples (data not shown). Overall, both natural whey starter cultures seemed to be characterized by high number of lactic acid bacteria, as previously reported [12,31]. A total of 100 colonies, 50 from each NWS, randomly chosen from the different selective media, were picked for further analysis. Among these, only 69 were successfully cultured and subjected to taxonomic analysis (Table S1).

### 3.2. Taxonomic Identification of Species Isolated from Natural Whey Starters Cultures

#### 3.2.1. Sequencing of V1–V3 rDNA Region

The use of highly variable V1-V3 rDNA region for the identification and detection of lactic acid bacteria has been generally accepted [17,28,32]. We performed taxonomic identification of the isolated colonies by analysis of the V1–V3 region. An amplicon of 520 bp was obtained from each isolate by using degenerate oligonucleotides complementary to conserved regions within the domain of Bacteria. PCR products were purified, sequenced, and subsequently analyzed by basic BLAST software. Sixty-nine clones were analyzed and the most significant results of the percent of V1–V3 sequence identity are reported in Supplementary Table S1. For many clones, the sequence analysis allowed the taxonomic identification of the genus but not of the species (Table S1). For this reason, we performed species-specific PCR to confirm the BLAST analysis.

#### 3.2.2. Species- and Subspecies-Specific PCR

**Figure 1:**
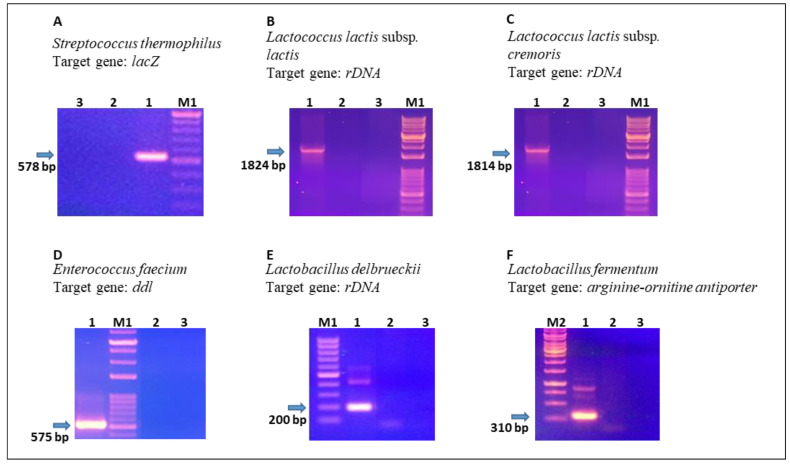
Amplification products obtained from the species/subspecies-specific PCR assay. An example of amplification product for each species isolated from NWSs is reported. In each panel lane 1 is loaded with the amplification product of the specific target gene and lanes 2 and 3 are negative controls. (**Panel A**). Lane 1: PCR product of amplified *lac Z* gene on *S. thermophilus* isolates; lanes 2 and 3: PCR on *L. lactis* DNA or without DNA, respectively. (**Panel B**). Lane 1: PCR product of amplified 16S rDNA (V1) and 16S/23S spacer region on *L. lactis* subsp. *lactis* isolates; lanes 2 and 3: PCR on *L. lactis* subsp. *cremoris* DNA or without DNA, respectively. (**Panel C**). Lane 1: PCR product of amplified 16S rDNA (V1) & 16S/23S spacer region on *L. lactis* subsp. *cremoris* isolates; lanes 2 and 3: PCR on *L. lactis* subsp. *lactis* DNA or without DNA, respectively. (**Panel D**). Lane 1: PCR product of amplified *ddl* gene on *E. faecium* isolates; lanes 2 and 3: PCR on *E. hirae* DNA or without DNA, respectively. (**Panel E**). Lane 1: PCR product of amplified 16S-23S rDNA spacer region on *L. delbrueckii* isolates; lanes 2 and 3: PCR on *L. plantarum* DNA or without DNA, respectively. (**Panel F**). Lane 1: PCR product of amplified arginine-ornitine antiporter gene on *L. fermentum* isolates; lanes 2 and 3: PCR on *L. plantarum* DNA or without DNA, respectively. M1: 1kb Opti-DNA (abm); M2: Hyper ladder 1 kb (Bioline).

As previously stated [26,33,34] and further confirmed by our blast analysis, it is not always possible to discriminate bacteria at the species level using 16S rDNA sequencing. Indeed, different techniques can be associated to make a selective identification at the species or subspecies level. We used a species- and subspecies-specific PCR approach targeting specific genes, which are less conserved than the 16S rDNA and have been demonstrated to be useful for the discrimination of closely related bacterial species. For this purpose, we targeted the following genes: *lacZ*, coding beta-galactosidase (*Streptococcus thermophilus*), 16S/23S rDNA region (*Lactococcus lactis* subsp. *cremoris*, *Lactococcus lactis* subsp *lactis*, and *Lactobacillus delbrueckii*), D-Ala/D-Ala ligase coding gene (*Enterococcus faecium*), and arginine-ornitine antiporter coding gene (*Lactobacillus fermentum*). These genes were chosen from those reported in the scientific literature and verified by in silico analysis of the sequences available on the NCBI (http://www.ncbi.nlm.nih.gov/sites/entrez, accessed on 17 November 2021). Some results of species-specific PCRs, as representative of the whole analysis, are shown in Figure 1.

The taxonomic analysis based on partial sequencing of the 16S rDNA and species/subspecies-specific PCR, allowed the identification of different species of LABs isolated from NWSc and NWSb. The following species were identified: *L. lactis* subsp. *lactis* (19 isolates), *L. fermentum* (1), *L. delbrueckii* (4), and *S. thermophilus* (4) from NWSb; *L. lactis* subsp. *lactis* (18 isolates), *E. faecium* (14), and *S. thermophilus* (9) from NWSc.

#### 3.2.3. 16S rDNA Next-Generation Sequencing Analysis (NGS)

**Figure 2:**
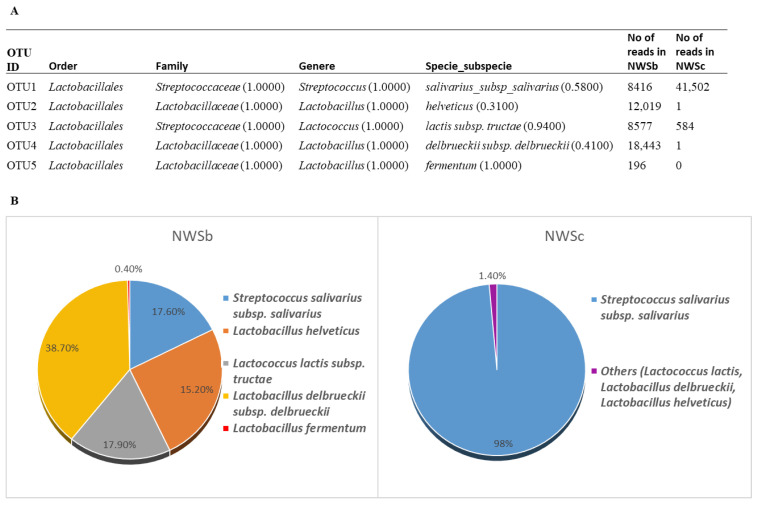
Metagenomic analysis of NWSs. (**A**). Taxonomic identification of OTUs and number of reads analyzed for each of them. Values in parentheses indicate statistical confidence of each taxonomic assignment, between 0 and 1 (with 1 higher reliability and 0.8 standard cut off). (**B**). Graphic representation of relative abundance of taxonomic units, based on the number of reads reported in panel A.

The taxonomic analysis of the two NWSs under investigation was further pursued by using the culture-independent metagenomic method. High-throughput 16S rDNA sequencing resulted in 83,256 and 56,697 raw reads obtained from NWSb and NWSc analysis, respectively. After the filtering step, analysis of 47,651(NWSb) and 42,088 (NWSc) reads revealed five OTUs (operational taxonomic unit), differently represented in the two NWSs (Figure 2). In both NWSs, the bacterial community is constituted by Firmicutes belonging to the order of *Lactobacillaceae*. Among those, the dominant genus detected in NWSb is *Lactobacillus*, with *L. delbrueckii*, *L. helveticus*, and *L. fermentum* species accounting for 38.7%, 25.2%, and 0.4% of the detected amplicons, respectively. *Lactococcus* and *Streptococcus* genera were also detected, namely *L. lactis* (17.9%) and *S. salivarius* species (17.6%). These results show some differences with those obtained with taxonomic analysis based on culture-dependent approaches (see above). In fact, the analysis conducted on only some colonies isolated from NWSb allowed the identification of four clones of *L. delbrueckii* (14% of identified colonies) and one clone of *L. fermentum* (4%), while *L. helveticus* was not detected. This apparent discrepancy could be due to the limitations of taxonomic analysis based on traditional culture-dependent approaches [35]. Markedly, less microbial diversity was found in the NWSc. Here, in fact, the NGS analysis identified *S. salivarius* as the dominant species, with 98% of amplicons, *Lc lactis* with 1.38% of amplicons and less than 1% of other *Lactobacillus* spp (*L. helveticus* and *L. delbrueckii*). Otherwise, taxonomic identification by culture-dependent methods showed a greater representativeness of lactococci (*L. lactis* subspp.) and enterococci (*E. faecium*) compared to streptococci (*S. thermophilus*). We also found a discrepancy between NGS and traditional culture methods results concerning the species of *Streptococcus*, which is annotated as *salivarius* (NGS) or *thermophilus* (culture-dependent molecular method). This is not surprising given that the 16S rDNA sequences exhibited almost no variability among streptococci, including *S. salivarius, S. vestibularis*, and *S. thermophilus* [36]. Indeed, the species-specific PCR used to confirm species of isolated colonies, was performed targeting the *lacZ* gene, specific for *S. thermophilus* identification (see Section 3.2.2). Moreover, we should consider that statistical analysis of NGS data shows that a good confidence value of the taxonomic results was obtained only for bacterial identification at genus level, while a less confident value characterized the results at the species and subspecies level (Figure 2). In any case, combining cultivation-dependent and cultivation-independent methods, four overlapping LAB species were found both in NWSb and NWSc, while one species was whey-specific (*L. fermentum* in NWSb and *E. faecium* in NWSc).

### 3.3. Optimization and Analysis of RAPD-PCR Profiles 

**Figure 3:**
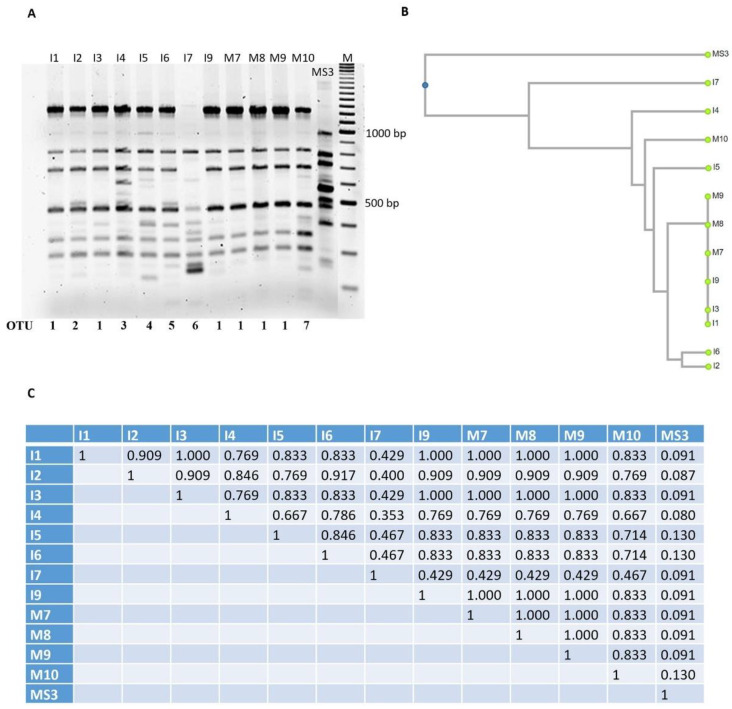
(**A**). RAPD patterns obtained with primer RAPD1 (see Mat and Met). RAPD-PCR products were obtained from the DNA of different clones of *L. lactis* subsp. *lactis* isolated from NWSc (I1, I2, I3, I5, I6, I9, M7, M8, M9, and M10). *L. lactis* subsp. *cremoris* MS3 (lab collection) was used as control. The patterns are grouped in clusters (OTU, Operational Taxonomic Units) according to the unweighted pair group method algorithm with arithmetic mean (UPGMA) analysis. (**B**). Dendrogram obtained from RAPD-PCR patterns, based on the Jaccard’s similarity coefficient, shown in panel (**C**). (**C**). Genetic similarity matrix of the analyzed clones, based on RAPD data and computed using Jaccard’s coefficient (UPGMA analysis).

**Figure 4:**
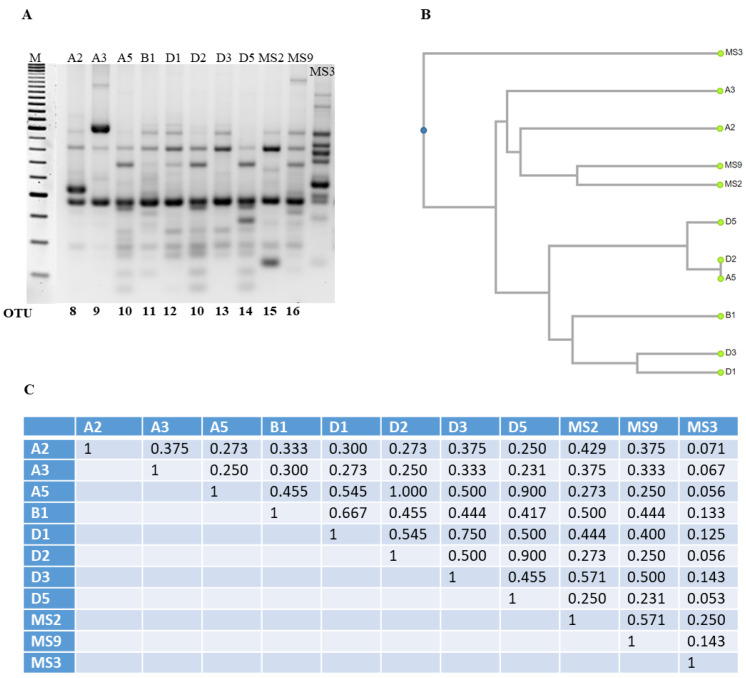
(**A**). RAPD patterns obtained with primer RAPD1. RAPD-PCR products were obtained from the DNA of different clones of *L. lactis* subsp. *lactis* isolated from NWSb (A2, A3, A5, B1, D1, D2, D3, D5, MS2, MS9, and MS3). *L. lactis* subsp. *cremoris* (lab collection) was used as control. The patterns are grouped in clusters (OTU, Operational Taxonomic Units) according to the unweighted pair group method algorithm with arithmetic mean (UPGMA) analysis. (**B**). Dendrogram obtained from RAPD-PCR patterns, based on the Jaccard’s similarity coefficient, shown in panel C. (**C**). Genetic similarity matrix of the analyzed clones, based on RAPD data and computed using Jaccard’s coefficient (UPGMA analysis).

The most abundant species found in both NWSb and NWSc, as revealed by the culture-dependent method, was *L. lactis* subsp. *lactis.* We tested the genetic diversity of this group of isolates by RAPD-PCR analysis. The reproducibility of fingerprint patterns and the discrimination efficiency of genotypic diversity were tested with different primers (RAPD1, RAPD2, XD9, and M13) among those described in the scientific literature (see Mat and Met) on a representative group of isolates of *L. lactis* subsp. *lactis* from NWSc (I1, I2, I3, I4, I5, I6, I7, I9, M7, M8, M9, and M10). RAPD2 and XD9 primers generated a lower number of bands respect to those generated by the M13 primer, although results obtained with M13 showed low reproducibility (data not shown). The best results were obtained with RAPD1, which generated high number of bands and good reproducibility. The data obtained by RAPD profiles of the above-mentioned isolates were clustered by UPGMA (unweighted pair group method algorithm with arithmetic mean) in seven OTU: OTU 1 included six isolates (I1, I3, I9, M7, M8, M9) with a similarity coefficient of 1.000, indicating that these isolates are representative of one single strain, while the rest included one isolate each: OTU 2 (I2), OTU 3 (I4), OTU 4 (I5), OTU 5 (I6), OTU 6 (I7), and OTU 7 (M10) (Figure 3). The OTUs 1, 2, 4, 5, and 7 share a similarity coefficient between 0.714 and 0.909. The lowest similarity was found for the OTU 3 and OTU 6. The latter shows a similarity coefficient of less than 0.500 with the other OTUs, indicating that the strain I7 is the one with a greater genetic diversity compared to the others (Figure 3). As expected, the lowest similarity was found for the MS3 strain, which is a *L. lactis* subsp. *cremoris* and was used as a control in the RAPD experiments. The same RAPD protocol with RAPD1 was applied to the *L. lactis* subsp. *lactis* isolated from NWSb, namely A2, A3, A5, B1, D1, D2, D3, D5, MS2, and MS9. Fingerprints of this bacterial group show patterns characterized by high variability with similarity coefficients less than 0.600 for all the isolates, except for A5 and D2, showing a similarity coefficient of 1.000, and therefore, representing the same strain (here named OTU 10), which shares a similarity coefficient of 0.9 with OTU 14 (D5). Finally, OTU 12 (D1) and OTU 13 (D3) show a similarity coefficient of 0.75. Hence, RAPD-PCR analysis of NWSb isolates established that the 10 analyzed isolates belong to nine different OTUs (Figure 4).

## 4. Discussion

Mozzarella and caciocavallo cheeses, traditionally produced in southern Italy, are often manufactured in small farms using the raw milk collected from allochthones animals. Some manufacturers still use natural whey starters (NWS) to maintain artisanal tradition during the processing of the raw milk. The microbial composition of NWS is determinant for the characteristic flavor and texture development to occur in artisanal cheese. The extensive microbial biodiversity of natural starter cultures has been demonstrated for different dairy products and, although characterized by the presence of specific LAB species, still presents a large variety among NWS and raw milk collected in the same geographical area, either at the level of species or at the level of strains [12,37]. The preservation of such specific biodiversity is fundamental for the maintenance of the major distinguishing traits of the final product. Studies on the identification of microbial strains of NWSs for various artisanal dairy products in different geographical areas have been extensively performed [7,8,10,12,13,14]. In general, the microbiota characterization aims at the possibility of periodically tracing the presence of specific LAB species and strains in NWSs for the maintenance of good quality and specific traits of the final product. The selected strains are usually also tested for putative metabolic and technological properties, since this market is always in search of new microbial strains which could improve the organoleptic and texture features of dairy products as well as their shelf life [38]. Moreover, the characterization of isolated strains from NWSs also aims at the search of new probiotic strains. In fact, today, the food market has an increasing request for products which, beside their nutritional value, may confer health benefit effects to the consumers. Evidence of the beneficial effects of some probiotic LAB is well provided in the scientific literature [39,40,41]. Health-promoting LAB strains have recently also been proposed for the production of postbiotics, namely water-soluble products deriving from bacterial metabolism or being by-products from bacterial cells after their lysis. Postbiotics represent last generation health promoting molecules of microbial derivation [42,43].

In this work, we study the microbial community of NWS cultures of cow’s milk for the production of caciocavallo and buffalo’s milk for the production of mozzarella, both from artisanal farms. Given the well-documented limitations encountered during conventional plating, frequently causing incomplete isolation and identification of microorganisms, we chose to apply culture-dependent and culture-independent techniques, with the aim to provide a global overview of the microbial composition. 

An overall picture of the presumptive lactic acid bacteria was determined by plate count on three different agar media (MRS, ESTY, and BHI) and by incubation at different temperatures (30–37–44 °C). Both NWS cultures analyzed were characterized by a high number of lactic acid bacteria. Overall, the microbial count of the different groups (lactobacilli, streptococci and lactococci, and enterococci) was higher in NWS from buffalo milk (ranging from 7.0 to 8.5 log CFU mL^−1^) than that found in NWS from cow milk (ranging from 4.7 to 6.6 log CFU mL^−1^). Moreover, while in NWSb all LAB groups were equally represented, in NWSc, lactobacilli were slightly less represented than streptococci, lactococci, and enterococci. A prevalence of lactococci compared to lactobacilli was also found in NWS cultures for the production of caciocavallo Silano PDO cheese [13] and Caciocavallo of Castelfranco cheese [44]. Indeed, the variability of the microbial composition of these starter cultures is linked to their own nature and to the process involving incubation of the whey drained from the cheese vat under environmental condition not rigorously controlled, especially in artisanal factories [12]. Several studies have characterized the microbiota of natural whey starter cultures used for the manufacturing of Italian mozzarella [12,17] and caciocavallo cheeses [13]. In those works, *S. thermophilus, L. lactis, L. garvie, L. helveticus, L. delbrueckii* subsp. *bulgaricus, L. fermentum, L. plantarum, L. casei*, and *E. faecalis* were frequently identified as dominant LAB in NWS for mozzarella production; lower microbial diversity was found in NWS for caciocavallo production, characterized mainly by thermophilic LABs, such as *S. thermophilus, L. helveticus*, and *L. delbrueckii*, even though the mesophilic *L. lactis* was also encountered. According to the microbial diversity described in the literature for NWS cultures, our results show higher microbial diversity in the NWSb compared to the NWSc. Despite the high number of presumptive lactobacilli detected by the culture-dependent method, only two species of lactobacillus were found in NWSb (*L. delbrueckii* and *L. fermentum*) and none in NWSc. This is probably due to the limitation of the cultivability of bacteria isolated from natural samples with high microbial diversity [45]. Therefore, to provide a global overview of the microbial composition, the taxonomic analysis of the two NWSs under investigation was also performed by the culture-independent metagenomic method. As expected, the NGS analysis revealed a microbial diversity only partially overlapping with the results obtained with culture-dependent methods. For instance, in both NWSs, *L. helveticus* was identified only by NGS analysis. Moreover, *L. lactis* subsp. *tructae* and *S. salivarius* were detected by NGS analysis, whereas *L. lactis* subsp. *lactis* and *S. thermophilus* were identified, respectively, among clones isolated by culturing and analyzed by 16S rDNA sequencing and species/subspecies-specific PCR. This is not surprising, given that the 16S rDNA sequences exhibit almost no variability among streptococci, including *S. salivarius, S. vestibularis*, and *S. thermophilus* [36]. Likewise, the high percent of identity also encountered for 16S rDNA sequences among *L. lactis* subspp. may account for the discrepancy in the identification of *tructae* (NGS) and *lactis* subspp (sequencing and subspecies-specific PCR) [46].

The NGS analysis performed on NWSc detected *S. salivarius* (or *S. thermophilus*, based on the above-mentioned observation) as a unique dominant species, according to the analysis of NWS for the production of traditional Caciocavallo cheese of Castelfranco [44].

Finally, NGS analysis did not detect the *E. faecium* species, which was isolated and identified by the cultivation approach. This may be due to the detection limits of the metagenomics approach, which could be affected by DNA isolation protocols [45]. These results outline the significance of applying both culture-dependent and -independent approaches for a more accurate species detection and identification of LAB in dairy products.

The high variety of rheology and flavor properties of artisanal cheeses, produced by microbial acidification, is due to the extreme variability of the microbial composition of the NWS cultures at species and strain level. In order to investigate the microbial diversity at biotype level, we performed RAPD-PCR fingerprinting of the *L. lactis* subsp. *lactis* isolates, which represent the most abundant species in both NWS under study. Based on the RAPD profiles, the 12 isolates from NWSc of *L. lactis* subsp. *lactis* were clustered in seven OTU, and the 10 isolates from NWSb were clustered in nine OTU. Overall, the results obtained by the genotypic clustering were in agreement with those obtained by classical identification procedures and NGS analysis, showing higher bacterial diversity of NWSb compared to NWSc at both species and strain level. Finally, data reported in this work confirm our previous results, obtained by analyzing the same NWS samples, showing LAB identification at genus, species, and subspecies level, by using-high resolution nano-LC-ESI MS/MS methodology [47].

## 5. Conclusions

Artisanal cheeses, such as mozzarella and caciocavallo, traditionally produced in the south of Italy, are mainly manufactured using NWS, whose complex microbiota gives rise to the characteristic flavor and texture of the final products. However, the natural variability of these starter cultures often does not guarantee the reproducibility and quality of the final product. In this contest, selection of LAB strains from natural whey starters aims to collect new starter bacteria to use for tracing microbial community during the production of artisanal cheeses, in order to preserve their quality and authenticity, and for selection of new LAB strains for the production of functional foods.

## Figures and Tables

**Table 1:** Primers used for species- and subspecies-specific PCR amplification.

Genus/Species/ Subspecies	Primer	Sequence	Target Gene	Amplicon Size (bp)	Ref.
*S. thermophilus*	Str-THER-F2116 Str-THER-R2693	GCTTGGTTCTGAGGGAAGC CTTTCTTCTGCACCGTATCCA	*lacZ*	578	[26]
*L. lactis* subsp. *lactis*	Lactis F 23SRevLac	GCTGAAGGTTGGTACTTGTA AGTGCCAAGGCATCCACC	*16S rRNA (V1) & 16S/23S spacer region*	1824	[27]
*L. lactis* subsp. *cremoris*	PLc(A)For 23SRevLac	GGTGCTTGCACCAATTTGAA AGTGCCAAGGCATCCACC	*16S rRNA (V1) & 16S/23S spacer region*	1814	[28]
*L. fermentum*	Lac-FER-F753 Lac-FER-R1062	CCAGATCAGCCAACTTCACA GGCAAACTTCAAGAGGACCA	*Arginine-ornitine antiporter*	310	[26]
*L. delbrueckii*	Del I Del II	ACGGATGGATGGAGAGCAG GCAAGTTTGTTCTTTCGAACTC	*16S-23S rRNA spacer region*	200	[29]
*E. faecium*	EfaeciumF EfaeciumR	GCAAGGCTTCTTAGAGA CATCGTGTAAGCTAACTTC	*D-Ala Ala ligase*	575	[27]

**Table 2:** Species- and subspecies-specific PCR conditions.

Genus/Species/Subspecies	Melting	Annealing	Elongation	Cycles Number
*S. thermophilus*	94 °C, 1 min	58 °C, 1 min	72 °C, 1 min	30
*Lc. lactis* subsp. *lactis*	94 °C, 1 min	58 °C, 1 min	72 °C, 2 min	30
*L. lactis* subsp. *cremoris*	94 °C, 1 min	60 °C, 1 min	72 °C, 2 min	30
*L. fermentum*	94 °C, 1 min	58 °C, 1 min	72 °C, 1 min	30
*L. delbrueckii*	95 °C, 30 s	55 °C, 30 s	72 °C, 30 s	30
*E. faecium*	94 °C, 1 min	54 °C, 1 min	72 °C, 1 min	30

**Table 3:** Cell numbers * (log CFU mL^−1^) of presumptive lactic acid bacteria groups found in the natural whey starter cultures (NWS) of cow or buffalo milk.

NWS Sample	Mesophilic Lactobacilli	Thermophilic Lactobacilli	Mesophilic Streptococci and Lactococci	Thermophilic Streptococci and Lactococci	Enterococci
Cow	5.6 ± 0.1	4.7 ± 0.1	6.6 ± 0.2	6.5 ± 0.1	6.5 ± 0.1
Buffalo	7.0 ± 0.2	8.5 ± 0.3	8.1 ± 0.1	7.5 ± 0.1	8.0 ± 0.2

* Mean values for two batches of each natural whey starter culture, analyzed in triplicate.

## Data Availability

This study did not report any data supporting reported results.

## Supplementary Materials

The following supporting information can be downloaded at: https://www.mdpi.com/article/10.3390/foods11020233/s1, Table S1: LAB isolated strains. The first three best results of V1-V3 sequence BLAST analysis are reported. Natural whey starter from cow (NWSc); natural whey starter from buffalo (NWSb); aerobiosis (AE); anaerobiosis (AN); Operational Taxonomic Unit (OTU); not applicable (na).
