# Supplementary material for: Isolation and Identification of Lactic Acid Bacteria from Natural Whey Cultures of Buffalo and Cow Milk
Source: Foods. 2022 Jan 16;11(2):233. doi: 10.3390/foods11020233 (PMC8774387; doi:10.3390/foods11020233)
Supplement: Supplementary file 1 [file foods-11-00233-s001.zip › foods-1493436-supplementary.pdf]

Table S1: LAB isolated strains. The first three best results of V1-V3 sequence BLAST analysis are reported. Natural whey starter from cow (NWSc); natural whey starter from buffalo (NWSb); aerobiosis (AE); anaerobiosis (AN); Operational Taxonomic Unit (OTU); not applicable (na).

ISOLATES	SOURCE/ MEDIUM-Growth conditions	V1-V3 SEQUENCE BLAST ANALYSIS	SPECIE/SUBSPECIE SPECIFIC PCR ANALYSIS	RAPD-PCR CLUSTERING
I1	NWSc/MRS 30°C-AE	Lactococcus lactis subsp. cremoris (98%)<br>Lactococcus lactis subsp. hordniae (98%)<br>Lactococcus lactis strain NBRC (98%)	*Lactococcus lactis subsp.lactis*	OTU 1
I2		Lactococcus lactis subsp. hordniae (98%)<br>Lactococcus lactis strain NBRC (98%)<br>Lactococcus lactis subsp. tructae (98%)	*Lactococcus lactis subsp.lactis*	OTU 2
I3		Lactococcus lactis subsp. cremoris (98%)<br>Lactococcus lactis subsp. hordniae (98%)<br>Lactococcus lactis strain NBRC (97%)	*Lactococcus lactis subsp.lactis*	OTU 1
I4		Lactococcus lactis subsp. cremoris (97%)<br>Lactococcus lactis subsp. hordniae (97%)<br>Lactococcus lactis strain NBRC (97%)	*Lactococcus lactis subsp.lactis*	OTU 3
I5		Lactococcus lactis subsp cremoris (98%)<br>Lactococcus lactis subsp hordniae (98%)<br>Lactococcus taiwanensis (98%)	*Lactococcus lactis subsp.lactis*	OTU 4
I6		Lactococcccus lactis strain NBRC (99%)<br>Lactococcus lactis strain NCD0 (99%)<br>Lactococcus lactis subsp hordniae (99%)	*Lactococcus lactis subsp.lactis*	OTU 5

I7		Lactococcus lactis strain NBRC (92%)<br>Lactococcus lactis strain NCDO (92%)<br>Lactococcus taiwanensis (96%)	*Lactococcus lactis subsp.lactis*	OTU 6
I8		Enterococcus faecium NBRC100485 (98%)<br>Enterococcus faecium NBRC 100486 (98%)<br>Enterococcus thailandicus (98%)	*Enterococcus faecium*	na
I9		Lactococcus lactis subsp cremoris (99%)<br>Lactococcus lactis subsp hordniae (99%)<br>Lactococcus lactis strain NBRC (99%)	*Lactococcus lactis subsp.lactis*	OTU 1
I10		Enterococcus faecium NBRC 100485 (96%)<br>Enterococcus faecium NBRC 100486 (96%)<br>Enterococcus thailandicus (96%)	*Enterococcus faecium*	na
L1	NWSc/MRS 30°C-AN	Lactococcus lactis strain NBRC (99%)<br>Lactococcus lactis strain NCDO (99%)<br>Lactococcus lactis subsp. hordniae (99%)	*Lactococcus lactis subsp.lactis*	na
L6		Lactococcus taiwanensis (99%)<br>Lactococcus lactis subsp. cremoris (99%)<br>Lactococcus lactis subsp. hordniae (99%)	*Lactococcus lactis subsp.lactis*	na
L7		Lactococcus taiwanensis (99%)<br>Lactococcus lactis subsp. cremoris (99%)<br>Lactococcus lactis subsp. hordniae (99%)	*Lactococcus lactis subsp.lactis*	na
L8		Lactococcus taiwanensis (99%)<br>Lactococcus lactis subsp. cremoris (99%)<br>Lactococcus lactis subsp. hordniae (99%)	*Lactococcus lactis subsp.lactis*	na
L9		Lactococcus taiwanensis (99%)<br>Lactococcus lactis subsp. cremoris (99%)<br>Lactococcus lactis subsp. hordniae (99%)	*Lactococcus lactis subsp.lactis*	na

L10		Lactococcus taiwanensis (99%)<br>Lactococcus lactis subsp. cremoris (99%)<br>Lactococcus lactis subsp. hordniae (99%)	*Lactococcus lactis subsp.lactis*	na
M1	NWSc/ESTY 30°C-AE	Enterococcus faecium NBRC100486 (97%)<br>Enterococcus faecium NBRC100485 (97%)<br>Enterococcus durans (97%)	*Enterococcus faecium*	na
M3		Enterococcus faecium NBRC100486 (97%)<br>Enterococcus faecium NBRC100485 (97%)<br>Enterococcus thailandicus (97%)	*Enterococcus faecium*	na
M5		Enterococcus faecium NBRC100486 (97%)<br>Enterococcus faecium NBRC100485 (97%)<br>Enterococcus thailandicus (97%)	*Enterococcus faecium*	na
M7		Lactococcus lactis cremoris (98%)<br>Lactococcus lactis hordniae (98%)<br>Lactococcus lactis strain NBRC (98%)	*Lactococcus lactis subsp.lactis*	OTU 1
M8		Lactococcus lactis subsp. cremoris (98%)<br>Lactococcus lactis subsp. hordniae (98%)<br>Lactococcus lactis subsp. NBRC (98%)	*Lactococcus lactis subsp.lactis*	OTU 1
M9		Lactococcus lactis subsp. cremoris (99%)<br>Lactococcus lactis subsp. hordniae (99%)<br>Lactococcus lactis strain NBRC (99%)	*Lactococcus lactis subsp.lactis*	OTU 1
M10		Lactococcus lactis subsp. cremoris (98%)<br>Lactococcus lactis subsp. hordniae (98%)<br>Lactococcus lactis strain NBRC (98%)	*Lactococcus lactis subsp.lactis*	OTU 1
N1	NWSc/ESTY 30°C-AN	Enterococcus faecium NBRC100486 (97%)<br>Enterococcus faecium NBRC100485 (97%)<br>Enterococcus thailandicus (97%)	*Enterococcus faecium*	na

N2		Enterococcus faecium NBRC100486 (96%)<br>Enterococcus faecium NBRC100485 (96%)<br>Enterococcus thailandicus (96%)	*Enterococcus faecium*	na
N3		Streptococcus thermophilus ATCC (97%)<br>Streptococcus thermophilus DSH (96%)<br>Streptococcus vestibularis (96%)	*Streptococcus thermophilus*	na
N5		Enterococcus hirae (99%)<br>Enterococcus faecium (99%)<br>Enterococcus hirae (99%)	*Enterococcus faecium*	na
Q1	NWSc/ESTY 44°C-AE	Streptococcus thermophilus (99%)<br>Streptococcus salivarius (99%)<br>Streptococcus thermophilus (98%)	*Streptococcus thermophilus*	na
Q2		Streptococcus thermophilus (99%)<br>Streptococcus salivarius (99%)<br>Streptococcus thermophilus (98%)	*Streptococcus thermophilus*	na
Q3		Streptococcus thermophilus (99%)<br>Streptococcus salivarius (99%)<br>Streptococcus thermophilus (98%)	*Streptococcus thermophilus*	na
Q4		Streptococcus thermophilus (99%)<br>Streptococcus vestibularis (94%)<br>Streptococcus salivarius (94%)	*Streptococcus thermophilus*	na
Q5		Streptococcus thermophilus (99%)<br>Streptococcus salivarius (99%)<br>Streptococcus vestibularis (99%)	*Streptococcus thermophilus*	na
Q6		Streptococcus thermophilus (99%)<br>Streptococcus salivarius (99%)<br>Streptococcus vestibularis (99%)	*Streptococcus thermophilus*	na

Q7		Streptococcus thermophilus (99%)<br>Streptococcus salivarius (99%)<br>Streptococcus vestibularis (99%)	*Streptococcus thermophilus*	na
Q10		Streptococcus thermophilus (99%)<br>Streptococcus salivarius (99%)<br>Streptococcus vestibularis (99%)	*Streptococcus thermophilus*	na
O3	NWSc/BHI<br>37°C-AE	Enterococcus faecium (98%)<br>Enterococcus hirae (98%)<br>Enterococcus faecium (97%)	*Enterococcus faecium*	na
O4		Enterococcus faecium (98%)<br>Enterococcus hirae (98%)<br>Enterococcus faecium (97%)	*Enterococcus faecium*	na
O5		Enterococcus faecium (98%)<br>Enterococcus hirae (98%)<br>Enterococcus faecium (97%)	*Enterococcus faecium*	na
O6		Enterococcus faecium (97%)<br>Enterococcus hirae (97%)<br>Enterococcus faecium (97%)	*Enterococcus faecium*	na
O7		Enterococcus faecium (98%)<br>Enterococcus faecium (98%)<br>Enterococcus hirae (97%)	*Enterococcus faecium*	na
O8		Enterococcus faecium (97%)<br>Enterococcus faecium (97%)<br>Enterococcus hirae (97%)	*Enterococcus faecium*	na
A2	NWSb/MRS<br>30°C-AE	Lactococcus lactis subsp. cremoris (93%)<br>Lactococcus lactis subsp. hordniae (93%)<br>Lactococcus lactis subsp. tructae (93%)	*Lactococcus lactis subsp.lactis*	OTU 8

A3		Lactococcus lactis (98%)<br>Lactococcus lactis (98%)<br>Lactococcus lactis subsp. hordniae (97%)	*Lactococcus lactis subsp.lactis*	OTU 9
A4		Lactococcus lactis subsp. cremoris 93%<br>Lactococcus lactis subsp. hordniae 93%<br>Lactococcus lactis strain NBRC 93%	*Lactococcus lactis subsp.lactis*	na
A5		Lactococcus lactis subsp. Hordniae (98%)<br>Lactococcus lactis subsp. hordniae (98%)<br>Lactococcus lactis subsp. tructe (98%)	*Lactococcus lactis subsp.lactis*	OTU 10
A8		Lactococcus lactis strain NBRC (96%<br>Lactococcus lactis strain NCDO 96%<br>Lactococcus lactis subsp. hordniae 96%	*Lactococcus lactis subsp.lactis*	na
A17		Lactococcus lactis (100%)<br>Lactococcus lactis (100%)<br>Lactococcus lactis subsp. hordniae (99%)	*Lactococcus lactis subsp.lactis*	na
A25		Lactococcus lactis (100%)<br>Lactococcus lactis (100%)<br>Lactococcus lactis subsp. hordniae (99%)	*Lactococcus lactis subsp.lactis*	na
A30		Lactococcus lactis strain NBRC (99%)<br>Lactococcus lactis strain NCDO (99%)<br>Lactococcus lactis subsp. hordniae (99%)	*Lactococcus lactis subsp.lactis*	na
A40		Lactococcus lactis subsp. cremoris (99%)<br>Lactococcus lactis subsp. hordniae (99%)<br>Lactococcus lactis (99%)	*Lactococcus lactis subsp.lactis*	na
A45		Lactococcus lactis subsp. hordniae (100%)<br>Lactococcus lactis (100%)<br>Lactococcus lactis subsp. tructae (100%)	*Lactococcus lactis subsp.lactis*	na

A48		Lactococcus lactis subsp. cremoris (99%)<br>Lactococcus lactis subsp. hordniae (99%)<br>Lactococcus lactis strain NBRC (99%)	*Lactococcus lactis subsp.lactis*	na
A50		Lactococcus lactis subsp. cremoris (100%)<br>Lactococcus lactis subsp. hordniae (100%)<br>Lactococcus lactis (100%)	*Lactococcus lactis subsp.lactis*	na
B1	NWSb/MRS 30°C-AN	Lactococcus lactis subsp. lactis (99%)<br>Lactococcus lactis subsp. lactis (98%)<br>Lactococcus lactis subsp. hordniae (97%)	*Lactococcus lactis subsp.lactis*	OTU 11
D1	NWSb/ESTY 30°C-AN	Lactococcus lactis subsp. lactis (99%)<br>Lactobacillus delbrueckii subsp. lactis (99%)<br>Lactococcus lactis subsp. cremoris (99%)	*Lactococcus lactis subsp.lactis*	OTU 12
D2		Lactococcus lactis subsp. Lactis (98%)<br>Lysinibacillus fusiformis (95%)<br>Lysinibacillus fusiformis (95%)	*Lactococcus lactis subsp.lactis*	OTU 10
D3		Lactococcus lactis subsp. Lactis (99%)<br>Lactobacillus delbrueckii subsp. lactis (99%)<br>Streptococcus sp. (92%)	*Lactococcus lactis subsp.lactis*	OTU 14
D4		Streptococcus thermophilus (99%)<br>Streptococcus sp. (97%)<br>Streptococcus salivarius (84%)	*Streptococcus thermophilus*	na
D5		Lactococcus lactis subsp. Lactis (93%)<br>Lactobacillus delbrueckii subsp. lactis (93%)<br>Streptococcus sp. (93%)	*Lactococcus lactis subsp.lactis*	OTU 15
MS2		Lactococcus lactis strain NBRC (99%)<br>Lactococcus lactis supsp. lactis (98%)<br>Lactococcus lactis subsp. hordniae (98%)	*Lactococcus lactis subsp.lactis*	OTU 16

MS9		Lactococcus lactis subsp. lactis (98%)<br>Lactococcus lactis subsp. hordniae (98%)<br>Lactococcus lactis strain NBRC (98%)	***Lactococcus lactis subsp.lactis***	OTU 17
E3	NWSb/MRS 44°C-AN	Streptococcus thermophilus (98%)<br>Streptococcus salivarius (97%)<br>Streptococcus thermophilus (97%)	*Streptococcus thermophilus*	na
E5		Streptococcus thermophilus (99%)<br>Streptococcus salivarius (98%)<br>Streptococcus thermophilus (98%)	*Streptococcus thermophilus*	na
E8		Streptococcus thermophilus (98%)<br>Streptococcus thermophilus (96%)<br>Streptococcus salivarius (92%)	*Streptococcus thermophilus*	na
E10		Lactobacillus fermentum (92%)<br>Lactobacillus fermentum (90%)<br>Lactobacillus sp. (90%)	*Lactobacillus fermentum*	na
F3	NWSb/ESTY 44°C-AE	Lactobacillus delbrueckii subsp.delbrueckii (98%)<br>Lactobacillus delbrueckii subsp.lactis (97%)<br>Lactobacillus delbrueckii subsp.bulgaricus (97%)	*Lactobacillus delbrueckii*	na
G1	NWSb/ESTY 44°C-AN	Lactobacillus delbrueckii subsp.bulgaricus (98%)<br>Lactobacillus delbrueckii subsp. indicus (98%)<br>Lactobacillus delbrueckii subsp.delbrueckii (97%)	*Lactobacillus delbrueckii*	na
G3		Lactobacillus delbrueckii subsp. bulgaricus (98%)<br>Lactobacillus delbrueckii subsp. delbrueckii (97%)<br>Lactobacillus leichmannii (94%)	*Lactobacillus delbrueckii*	na
G5		Lactobacillus delbrueckii subsp. bulgaricus (98%)<br>Lactobacillus delbrueckii subsp. indicus (98%)<br>Lactobacillus delbrueckii subsp. delbrueckii (97%)	*Lactobacillus delbrueckii*	na
